# Heterocyclic Schiff Bases of 3-Aminobenzanthrone and Their Reduced Analogues: Synthesis, Properties and Spectroscopy

**DOI:** 10.3390/molecules26092570

**Published:** 2021-04-28

**Authors:** Natalja Orlova, Irena Nikolajeva, Aleksandrs Pučkins, Sergey Belyakov, Elena Kirilova

**Affiliations:** 1 Laboratory of Physical Organic Chemistry, Latvian Institute of Organic Synthesis, LV-1006 Riga, Latvia; natalja_orlova@inbox.lv (N.O.); serg@osi.lv (S.B.); 2 Department of Applied Chemistry, Institute of Life Sciences and Technology, Daugavpils University, LV-5401 Daugavpils, Latvia; irena.nikolajeva@grindeks.lv (I.N.); aleksandrs.puckins@du.lv (A.P.)

**Keywords:** Schiff bases, benzanthrone, heterocycles, reduction, fluorescence spectroscopy

## Abstract

New substituted azomethines of benzanthrone with heterocyclic substituents were synthesized by condensation reaction of 3-aminobenzo[de]anthracen-7-one with appropriate aromatic aldehydes. The resulting imines were reduced with sodium borohydride to the corresponding amines, the luminescence of which is more pronounced in comparison with the initial azomethines. The novel benzanthrone derivatives were characterized by NMR, IR, MS, UV/Vis, and fluorescence spectroscopy. The structure of three dyes was studied by the X-ray single crystal structure analysis. The solvent effect on photophysical behaviors of synthesized imines and amines was investigated. The obtained compounds absorb at 420–525 nm, have relatively large Stokes shifts (up to 150 nm in ethanol), and emit at 500–660 nm. The results testify that emission of the studied compounds is sensitive to the solvent polarity, exhibiting negative fluorosolvatochromism for the synthesized azomethines and positive fluorosolvatochromism for the obtained amines. The results obtained indicate that the synthesized compounds are promising as luminescent dyes.

## 1. Introduction

Azomethines, imines, anils, or Schiff bases are useful intermediates in the synthesis of important pharmaceutical and biochemical substances due to multifunctional transformations via reductions, condensations, additions, etc. [1]. These substances have the great potentials to be used in various fields such as electrochemistry, catalysis, optical devices, pharmaceutical industry, and in environmental chemistry [2,3,4]. Up to now, various synthesis methods have been reported, such as the dimerization, oxidation, or dehydrogenation of primary amines, and the coupling alcohols and amines [5,6]. Traditionally, the condensation of active aldehydes and amines is the most common and efficient way to obtain imines due to the easy availability of initial amines.

Many heterocyclic Schiff bases show promising physical, chemical, and biological properties. An interest in the exploration of novel heteroaromatic azomethines has undoubtedly been growing due to their proven usefulness as attractive lead structures for the development of catalysts, intermediates in organic synthesis, dyes [7], pigments, polymer stabilizers [8], and corrosion inhibitors [9]. For example, a number of heteroaromatic imines containing a pyridine, thiophene, or furan ring were synthesized [10], demonstrating optical and electrochemical responses towards the acid environment. Polyimines containing a pyridine ring showed multichromatic emissions in various solvents [11]. Heterocyclic azomethines, which are ion-active “naked-eye” switches of spectral properties in the presence of fluorine, acetate, and dihydrophosphate ions, are described by Popova et al. [12]. Some of the heteroaromatic imines can act as chemosensors for cations [13].

A variety of azomethine derivatives bearing heterocyclic ring have been known to show a wide range of biological activities including antioxidant [14], antibacterial [3], antifungal [15], analgesic [16], and antitumor [17] activity.

Today, luminescent benzanthrone derivatives are used in various fields, such as synthetic and natural fiber dyes, photoconductors, luminophores, fluorescent labels, and probes [18]. The first literature data on benzanthrone azomethines have been published since the 1990’s. First reported azomethines of 3-aminobenzantrone are described in papers [19,20], the authors of which consider these compounds to be promising as luminescent dyes for liquid crystals and polymer media. The molecular structure of these dyes sterically corresponds to the structure of liquid crystal molecules, as in compounds of the guest–host type. Due to their regular arrangement and appropriate phase transitions, they can be used in nematic liquid crystal matrices for various technological solutions [20,21]. 3-Aminobenzanthrone azomethines are able to form charge-transfer complexes with iodine [22] and also with metals, similar to other aromatic azomethines [23]. These properties may be used for the iodine determination by optical chemical sensors. Sensor performance and iodine detection are based on quenching the fluorescence of 3-aminobenzanthrone azomethines in the presence of iodine [24].

Despite the previously discovered interesting properties of 3-iminobenzantrone, only a few benzaldehyde derivatives have been synthesized and described in the literature. Benzanthrone azomethines with heterocyclic substituents are not described in the literature at all, but may have interesting physical and chemical properties.

In our previous investigations, it was demonstrated that various nitrogen-containing derivatives of benzanthrone have various optical properties with emission in the spectral region from green to red [25,26,27,28]. Based on the previous research and following our current interest in the synthesis of emissive benzanthrone dyes containing the azomethine group, there will be reported research about the synthesis and characterization of a series of heterocyclic Schiff bases and the possibility of their reduction to the corresponding amines. The obtained compounds were fully characterized, including the determination of the structure by single crystal X-ray diffraction analysis and photophysical parameters in various media.

## 2. Results and Discussion

### 2.1. Synthesis and Characterization of Azomethines

Since imines are of great interest for the development of new materials, and on the other hand, Schiff bases are relatively rarely used as fluorescent dyes, we decided to prepare such benzanthrone derivatives. Considering our experience in the synthesis of amino, amido, amidino, and azo derivatives of benzanthrone and the good optical properties of them, it was of interest to synthesize new Schiff bases by introducing an azomethine group into the benzanthrone molecule, expanding the system of conjugated double bonds and, thus, increasing the chromophore length. As a result, substances with new photophysical properties can be obtained.

**Scheme 1:**
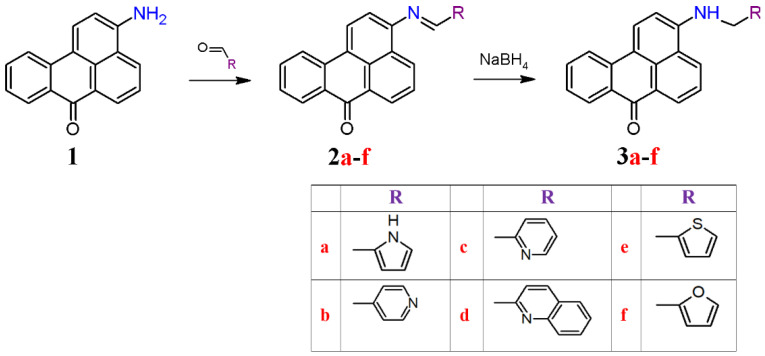
Synthetic route for the preparation and reduction of azomethines.

It is known that the condensation of appropriate carbonyl compounds with primary amines is the most common and efficient method for preparation of imines. The target derivatives (**2a**–**f**) investigated in the present study have been synthesized by condensation of amine **1** with corresponding heterocyclic aldehydes (see Scheme 1).

In previous studies, Grabchev and co-workers [19,20] described the synthesis of benzanthrone azomethines, which was carried out by heating 3-aminobenzanthrone with aromatic aldehyde in excess or by boiling in ethanol for 6 h. However, we used another solvent (toluene); thus, it was possible to increase the reaction temperature to 110 °C compared to the reaction in ethanol. The main problem during the synthesis was water formation and its separation from the reaction medium, since imines can be hydrolyzed in the presence of water. For water binding, 4 Å molecular sieves were added to the reaction mixture. Therefore, the synthesis of target azomethines was realized by heating 3-aminobenzanthrone with corresponding heterocyclic aldehyde in the boiling toluene. The imines were obtained with 65–90% yields as crystalline compounds, which had colors from yellow to brown.

The identification of prepared substances was realized using IR and NMR spectra. The obtained spectroscopic data fully confirm the structures of the synthesized imines. The infrared spectra of the obtained compounds have characteristic absorption bands: 1592–1647 cm^−1^, which corresponds to a conjugated C=N bond; C=O (1640–1668 cm^−1^), C=C (1512–1574 cm^−1^), as well as fluctuations of the C–H bond (2924–3073 cm^−1^).

**Figure 1:**
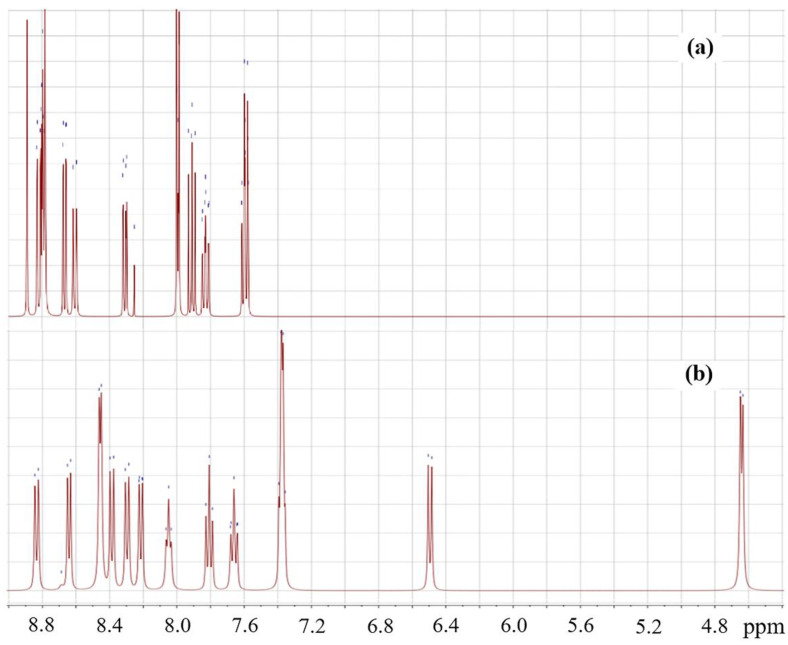
^1^H-NMR spectra of derivatives **2b** (**a**) and **3b** (**b**) in d6-DMSO solution.

The ^1^H NMR spectra clearly show a typical multiplet signal of aromatic protons at 6.20–9.00 ppm (see Figure 1). This multiplet consists of nine protons of the benzanthrone residue, 3–6 heterocyclic protons, and an additional proton of the azomethine group, which, as a singlet, is located at 8.55–8.95 ppm. The NH group of pyrrole residue (in imine **2a**) demonstrates singlet signal in a weak field at 12.01 ppm.

In ^13^C NMR spectra carbonyl group demonstrates typical signal about 182–190 ppm, but aromatic carbon atoms show multiple signals at 110–150 ppm.

In the mass spectra of the derivatives obtained, the molecular ion and in some cases ions with M+1 and/or M−1 were detected, except for the spectra of derivatives with quinoline (**2d**) and furan (**2e**) residues, where the molecular ion was not detected, probably due to low stability of these derivatives. The first peak was identified as 3-aminobenzanthrone ion (*m/z* 245) in the spectra of these compounds.

The ion with a mass of 256 is observed in the mass spectra of azomethines, in contrast to the previously studied amino and amidino derivatives [25,27]. The presence of this peak indicates that the fragmentation of the molecular ion occurs through the cleavage of the bond between the imino group and the heterocycle, with the further formation of the isonitrile ion (*m/z* 256), and then the isonitrile ion with further fragmentation to 1-phenylnaphthalene (M = 201) ion, which is usual for benzanthrone derivatives losing C=O and side chains [25,27].

**Figure 2:**
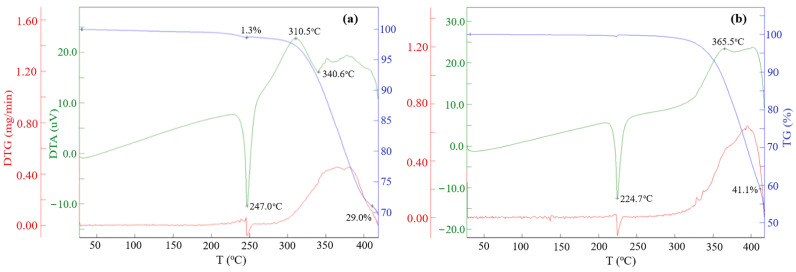
TG, DTG, DTA, and temperature curves as a function of time for derivatives **2a** (**a**) and **3a** (**b**), heating rate 10 °C min^−1^, air.

Thermal analysis data demonstrate that the obtained compound is stable up to 300 °C (5% weight loss). At higher temperatures, the substance begins to lose weight rapidly (see Figure 2).

Some of the obtained azomethines have a low stability in a water-containing medium undergoing hydrolysis that produces initial amine. This property is an essential disadvantage for using imines as fluorescent probes for biomolecules investigation in the water environment. Furthermore, these dyes exhibit a relatively low intensity green-yellow fluorescence. Despite the low stability and tendency to hydrolysis, some benzanthrone azomethines have been proposed for use in various fields. Thus, azomethines derived from substituted benzaldehydes have been studied as luminophore dyes in liquid crystal systems for electro-optical displays and for polymer films [19,20,21]. Covalently immobilized aminobenzanthrone Schiff base can be applied as an optical chemical sensor for iodine [24]. Recently, imine derived from salicylic aldehyde has been used for capturing environmentally hazardous metal ions [29].

### 2.2. Reduction of Obtained Azomethines

The chemical transformations of imines are of great interest, since a number of useful compounds can be obtained as a result of these reactions. In particular, new fluorescent fused heterocyclic derivatives of anthraquinoline were obtained in the cyclization reaction of benzanthrone azomethines with 3-oxo-butyric acid ester [30]. One of the main imine properties is the ability to be reduced to the corresponding amine. However, similar reactions for benzanthrone azomethines are not described in the literature. Therefore, the conditions and reagents for the reduction of the C=N bond in the synthesized azomethines were studied in the present work.

Catalytic hydrogenation (palladium on carbon, Renay nickel) [31] and reduction with the metal hydrides and borohydrides [32] are commonly used for efficient imine reduction. Attempts to reduce the resulting imines using hydrogen over platinum or Raney nickel were unsuccessful. Another tested reduction method is the treatment of the imine with zinc in glacial acetic acid. It was found by thin-layer chromatography that products with an orange glow were formed. However, it was not possible to isolate the product from the impurities with a blue glow. An undivided mixture was also obtained in the reaction of imines with lithium aluminum hydride in alcohols.

Sodium borohydride NaBH_4_ is known as a cheap and environmentally friendly reagent. The reduction of azomethines was carried out in dimethylformamide with finely ground NaBH_4_, since the imines of 3-aminobenzanthrone are readily soluble in DMF. Methanol is used as the reaction catalyst. NaBH_4_ ionizes in methyl alcohol, and the negatively charged -BH_4_ ion reacts with imine. During the reaction, NaBH_3_(OMe), NaBH_2_(OMe)_2_, and NaBH(OMe)_3_ are formed, which are also reducing agents, but weaker than NaBH_4_. As a result, one mole of NaBH4 is able to reduce four moles of the corresponding imine. Since imines are rapidly hydrolyzed, a four-fold excess of the reducing agent was used in the reduction reaction to make the reduction as fast as possible, and the azomethine did not have time to convert to the unmodified amine. The reaction yield of 3-amino derivatives (**3a**–**f**) reaches 70–78%. The resulting derivatives **3a**–**f** are bright orange or red colored compounds with pronounced luminescent properties in organic solvents.

The obtained IR and NMR spectroscopic data of prepared confirm the structures of the synthesized amino derivatives. The IR spectra show the oscillations of newly formed N–H bonds at 3350–3450 cm^−1^. As can be seen from the ^1^H NMR spectrum of amine **3b** (Figure 1), the characteristic azomethine **2b** proton singlet at 8.85 ppm disappears, but a doublet signal of the formed CH_2_ group appears at 4.64 ppm. The broad triplet signal occurs at 8.05 ppm, which belongs to the disubstituted amino group. The signal of the proton at position 2 of the benzanthrone system is shifted significantly upfield (the doublet at 6.50 ppm) due to the changed electron density on the nitrogen atom.

Analyzing the DTA/TG curve (see Figure 2 for compounds **2a** and **3a**), it can be concluded that amino derivatives are slightly more thermally stable than azomethines, since amines lose 5% of their weight at about 350 °C, in comparison with azomethines, which lose 5% of their weight up to 300 °C.

### 2.3. X-ray Crystallographic Study

The structures of 3-[N-(1H-pyrrol-2-ylmethylidene)amino]benzo[*de*]anthracen-7-one (**2a**), 3-[N-(thiophen-2-ylmethylidene)amino]benzo[*de*]anthracen-7-one (**2e**) and 3-[N-(1H-pyrrol-2-ylmethyl)amino]benzo[*de*]anthracen-7-one (**3a**) were also confirmed by X-ray diffraction analysis. The studied single crystals were grown in benzene. The data obtained fully confirm that the aromatic part of the benzanthrone molecule is flat.

**Figure 3:**
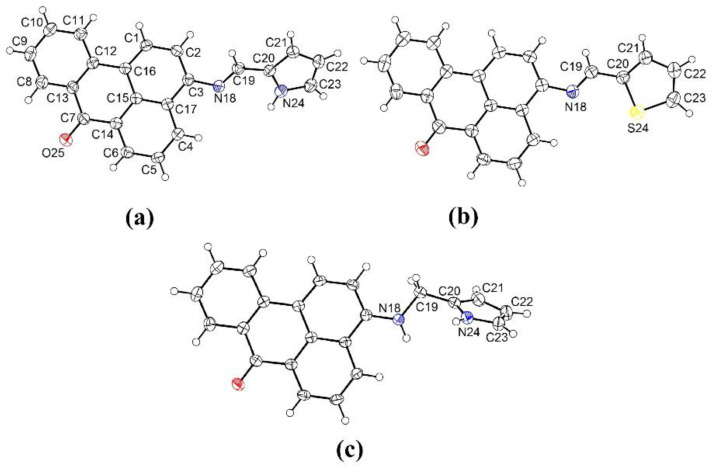
ORTEP diagram of molecules **2a** (**a**), **2e** (**b**), and **3a** (**c**). Atomic numbering scheme of the benzanthrone systems in **2e** and **3a** corresponds to one in **2a**.

Figure 3 illustrates ORTEP diagrams of molecules **2a**, **2e**, and **3a** showing the labeling scheme followed in the text and thermal displacement ellipsoids at the 50% probability level for non-hydrogen atoms. Table 1 lists the main bond lengths and valence angles for heterocycles and heteroatoms of **2a**, **2e**, and **3a**.

The values of dihedral angles between heterocycles and benzanthrone system are 44.4, 17.7, and 68.9° for **2a**, **2e**, and **3a**, respectively. The presence of a methylene group in **3a** disrupts conjugation between the heterocycle and benzanthrone. A high value of the torsion angle confirms the absence of conjugation. In the molecular structure of **2e**, there is the intramolecular σ-hole interaction between atoms of N18 and S24. The N18···S24 distance is equal to 3.041(1) Å. By means of this interaction, the structure does not exhibit the structural disorder that is so characteristic of compounds containing a thiophen-2-yl fragment [33].

In the crystal structure of **2a**, there is found a quite strong intermolecular hydrogen bond of NH⋅⋅⋅O type with length of 2.883(1) Å (distance O25⋅⋅⋅H24 is 1.96(2) Å, angle O25⋅⋅⋅H24−N24 is 162(1)°). In the crystals, by means of this bond, the molecular chains are formed along the monoclinic axis. Additionally, there is a weak intermolecular π–π stacking interaction between benzanthrone systems (interplanar distance is 3.528(3) Å) in the crystal structure. 

The crystal structure of **2e** is characterized by a strong intermolecular π–π stacking interaction between benzanthrones. The interplanar distance between the planes of benzanthrones is 3.410(4) Å, but the shortest intermolecular atom–atomic distance is the contact of C3⋅⋅⋅C7 with 3.354(2) Å. By means of these interactions the centrosymmetric molecular dimers are formed in the crystal structure.

In the crystal structure of **3a** a solvent (benzene) molecule has been found. The benzene molecules lie in the special positions (in the centers of inversion), while the molecules of benzanthrone derivative are in general positions. Thus, there are two molecules of the benzanthrone derivative per benzene molecule; therefore, the substance **3a** represents a semisolvate. In the crystals, there are found strong intermolecular hydrogen bonds of NH⋅⋅⋅O type between carbonyl groups and NH-groups of pyrrole fragment. The parameters of these bonds are following: O25⋅⋅⋅N24 = 2.855(1) Å, O25⋅⋅⋅H24 = 1.92(2) Å, and O25⋅⋅⋅H24−N24 = 169(1)°.

**Figure 4:**
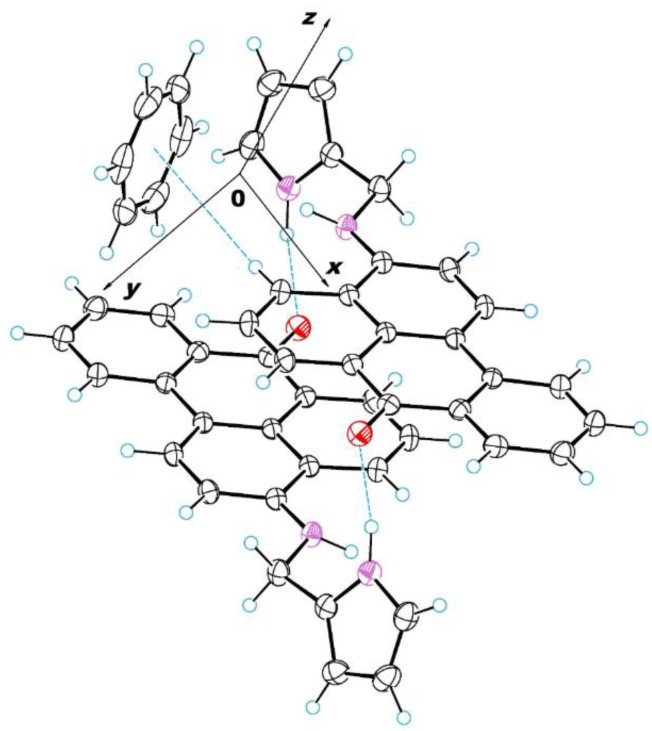
A fragment of molecular packing of **3a** showing N−H⋅⋅⋅O and C−H⋅⋅⋅π hydrogen bonds.

The centrosymmetric molecular dimers are formed by means of the hydrogen bonds in the crystal structure. These dimers are also connected by a π–π stacking interaction: the distance between benzanthrone planes is 3.411(3), but the shortest atom–atomic contact (C3⋅⋅⋅C6) is equal to 3.385(2) Å. A weak hydrophobic interaction C−H⋅⋅⋅π (distance H⋅⋅⋅π = 3.20 Å) between C4−H4 and benzene molecule can also be noted. Figure 4 gives a fragment of molecular packing of **3a**.

### 2.4. Spectroscopic Properties

**Figure 5:**
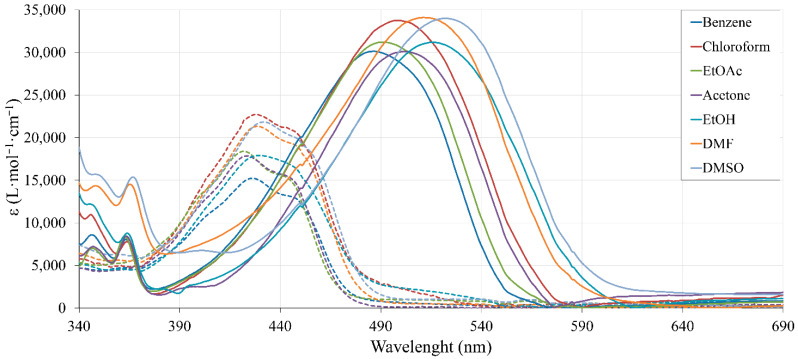
The absorption spectra of azomethine **2c** (dashed lines) and amine **3c** in various organic solvents.

**Figure 6:**
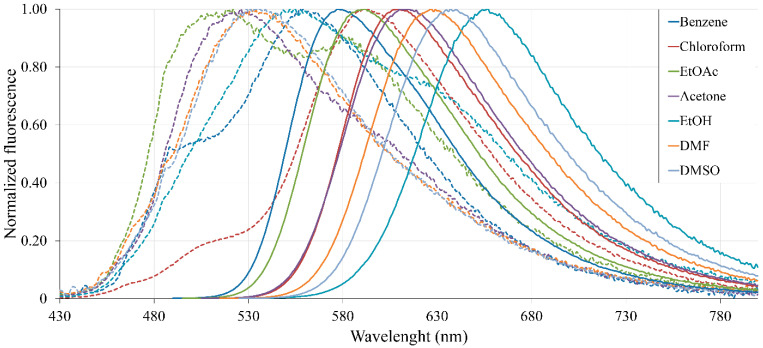
The emission spectra of azomethine **2c** (dashed lines) and amine **3c** in various organic solvents (concentration 10^−5^ M).

The photophysical properties of the synthesized azomethines and amines were evaluated, and the corresponding data are summarized in Table 2, Table 3 and Table 4. The absorption and emission spectra were recorded in eight organic solvents with a wide range of polarities. The absorption and emission spectra for one of the pairs of azomethine and amine (compounds **2c** and **3c**) are shown in Figure 5 and Figure 6. For benzanthrone 3-substituted derivatives, the light absorption and emission properties depend mainly on the electron donor–acceptor interaction between the group in the 3-position and the carbonyl group through the conjugated part of the molecule [34]. The starting 3-aminobenzanthrone is characterized by absorption at 460–530 nm and by luminescence at 560–670 nm and is used as an orange-red glow phosphor [35].

The spectral characteristics of the newly obtained imines differ greatly from the initial amine **1**, when the amino group is replaced by the azomethine group, since the electron-donating ability of the azomethine moiety for the obtained compounds **2a**–**f** is significantly reduced compared to the starting amine **1**. As a result, the absorption peak is hypsochromically shifted by 40–80 nm, showing a long-wavelength band at 420–450 nm (with a molar extinction coefficient of about 20,000 M^−1^cm^−1^), the position of which is practically independent of the solvent polarity.

The absorption spectra of the synthesized amines **3a**–**f** have an absorption band at 360–380 nm and a second broad intense absorption band at 490–540 nm with a molar absorptivity of ~30,000 M^−1^cm^−1^.The synthesized azomethines show two-band emission, especially pronounced for the furan derivative. A physical interpretation of this phenomenon was given using the twisted state model (the TICT state), according to which the short-wavelength band in the fluorescence spectrum is due to the existence of molecules with a planar configuration, and the emission in the long-wavelength region is determined by molecules in which the planes of the donor and acceptor parts are mutually perpendicular [36]. The formation of the TICT state was also detected in a previous study of the photophysical properties of 3-amino derivatives of benzanthrone [28,37]. The very low luminescence efficiency also confirms the formation of the TICT state, as this indicates nonradiative decay through twisted intramolecular charge transfer.

The emission spectra of the synthesized amines have maxima at 530–660 nm, depending on the solvent. In addition, the luminescence efficiency of amines is 8–10 times higher. The studied azomethines mainly exhibit a slight negative fluorosolvatochromism due to the polarity-induced inversion of ππ* and nπ* states in derivatives with electron-withdrawing groups [38]. However, amino derivatives show a large positive fluorosolvatochromism and significant Stokes’s shift (~ 90–120 nm). Thus, the light absorption and luminescence parameters of the synthesized amino derivatives depend much more on the polarity of the solvent than the starting azomethines. This means that these compounds have a pronounced tendency toward intramolecular charge transfer (ICT). Such compounds exhibit different properties in the ground and excited states. The difference in energy between the Frank–Condon state and the ICT state is responsible for the large Stokes shift, which is preferred in most fluorescence methods due to the reduced chromatic aberration and increased resolution in bioimaging applications [39,40].

## 3. Experimental Method

### 3.1. Materials and Basic Measurements

All reagents were of analytical grade (Aldrich Chemical Company, Munich, Germany) and were used as received. The progress of the chemical reactions and the purity of products were monitored by TLC on silica gel plates (Fluka F60254, 20 × 10, 0.2 mm, ready-to-use), using with C_6_H_6_-CH_3_CN (3:1) as eluent and visualization under UV light. Column chromatography on silica gel was carried out on a Merck Kieselgel (230–240 mesh) with dichloromethane as eluent. Melting points were determined on an MP70 Melting Point System apparatus and are not corrected.

The identification of the chemical bonds was performed by means of Fourier-transform infrared (FTIR) spectrometry. A Bruker Vertex 70v vacuum spectrometer equipped with an attenuated total reflection (ATR) accessory was used in this study. At least three spectra per sample were measured with a recording range 400–4000 cm^−1^, spectral resolution ± 2 cm^−1^, and in vacuum 2.95 hPa, and the average spectrum was calculated from the measured spectra. ^1^H NMR spectra were recorded on Bruker equipment, operating at 400 MHz in CDCl_3_ or DMSO-d_6_ (with TMS as internal standard) at ambient temperature. ^13^C NMR spectra were run in the same instrument at 100 MHz using the solvent peak as internal reference.

A Shimadzu GCMS-QP2010 system (Shimadzu Corporation, KYOTO, Japan) was used for the analysis and mass spectra were recording. The gas chromatograph was equipped with an electronically controlled split/splitless injection port. GC was carried out on a 5% diphenyl-/95% dimethylpolysiloxane fused-silica capillary column (Rtx-5SIL-MS, 30 m × 0.32 mm, 0.25 µm film thickness; Restek). Helium (99.999%) was used as the carrier gas at a constant flow of 1.6 mL min^−1^. The injection (injection volume of 1 µL) was performed at 250 °C in the split mode, with the split ratio 1:10. The oven temperature program was as follows: the temperature was held at 30 °C for 5 min, then 30–180 °C at the rate of 10 °C min^−1^, 180–300 °C at the rate of 15 °C min^−1^, and finally held at 300 °C for 5 min. The mass spectrometer was operated in the electron ionization mode (ionization energy of 70 eV). The source and transfer line temperatures were 200 and 310 °C, respectively. Detection was carried out in the scan mode: *m/z* 35–500.

Simultaneous TG-DTA and DSC curves were collected using a Exstar6000 TG/DTA 6300 thermal analyzer with a heating rate of 10 K min^−1^ in temperature interval 30–400 °C with samples masses of approximately 5 mg. Aluminum crucibles were used for analysis.

All commercial reagents were of analytical grade (Aldrich Chemical Company) and were used as received. 3-Aminobenzanthrone (**1**) was prepared by nitration of benzanthrone and subsequent reduction of the obtained 3-nitroderivative according to the literature procedure [41].3.2. Synthesis and Characterization

General Procedure for synthesis of azomethines **2a**–**f**: Compound **1** and the corresponding aldehyde were placed in a round-bottom flask, at a ratio of 1:2. Then, 10–15 mL of dry toluene and molecular sieves 4 A were added. The reaction mixture was heated to reflux and stirred for 8–10 h. The reaction mixture was cooled to room temperature, precipitated with diethyl ether. The resulting precipitate was filtered off and washed with methanol, recrystallized from benzene, and dried.

**3-[N-(1*H*-Pyrrol-2-ylmethylidene)amino]benzo[*de*]anthracen-7-one (2a).** The product was obtained as an orange crystalline solid with a yield of 77%; mp 247-248 °C; ^1^H NMR (400 MHz, DMSO) δ 12.01 (1H, s, NH), 8.99 (1H, dd, *J* = 8.0, 1.1 Hz), 8.72 (1H, d, *J* = 8.0 Hz), 8.66 (1H, dd, *J* = 6.7, 1.1 Hz), 8.57 (1H, d, *J* = 8.0 Hz), 8.55 (1H, s), 8.31 (1H, dd, *J* = 7.8, 1.1 Hz), 7.89 (1H, t, *J* = 7.8 Hz), 7.82 (1H, td, *J* = 7.5, 1.1 Hz), 7.57 (1H, t, *J* = 7.5 Hz), 7.46 (1H, d, *J* = 8.0 Hz), 7.17 (1H, s), 6.85 (1H, d, *J* = 4.2 Hz), 6.28 (1H, d, *J* = 6.7 Hz); ^13^C NMR (100 MHz, DMSO) δ 183.2, 151.7, 151.5, 136.5, 134.3, 132.4, 131.4, 130.2, 130.1, 129.2, 128.3, 128.0, 127.7, 127.1,126.6, 125.5, 124.1, 122.9, 118.3, 114.3, 110.7; FTIR (KBr) ν_max_ 3298, 3044, 1640, 1610, 1564, 1505, 1458, 1382, 1302, 1280, 1086, 1042, 970, 856, 780, 734, 602 cm^-1^ EIMS *m/z* 322 [M]^+^ (89), 321 (100), 292 (14), 265 (8), 239 (6), 227 (12), 201 (18), 200 (22), 161 (14), 147 (16).

**3-[N-(Pyridin-4-ylmethylidene)amino]benzo[*de*]anthracen-7-one (2b).** The product was obtained as an orange crystalline solid with a yield of 77%; mp 260–261 °C; ^1^H NMR (400 MHz, DMSO) δ 8.95 (1H, s), 8.82–8.90 (4H, m), 8.72 (1H, dd, *J* = 7.0, 1.2 Hz), 8.66 (1H, d, *J* = 7.8 Hz), 8.36 (1H, dd, *J* = 7.8, 1.6 Hz), 8.02–8.07 (2H, m), 7.97 (1H, t, *J* = 7.8 Hz), 7.89 (1H, td, *J* = 7.8, 1.6 Hz), 7.62-7.68 (2H, m); ^13^C NMR (100 MHz, DMSO) δ 189.3, 158.9, 150.9, 149.6, 142.5, 135.9, 133.4, 131.1, 130.7, 130.4, 128.4, 128.3, 128.1, 124.6, 122.8, 122.4, 113.5; FTIR (KBr) ν_max_ 3028, 1640, 1595, 1569, 1382, 1317, 1280, 1218, 1084, 839, 781, 746, 701 cm^-1^; EIMS *m/z* 334 [M]^+^ (100), 256 (16), 202 (12), 201 (27), 200 (25).

**3-[N-(Pyridin-2-ylmethylidene)amino]benzo[*de*]anthracen-7-one (2c).** The product was obtained as a yellow powder with a yield of 75%; mp 230–231 °C; ^1^H NMR (400 MHz, CDCl3) δ 8.82 (1H, dd, *J* = 7.4, 1.2 Hz); 8.78 (1H, s); 8.66 (1H, dd, *J* = 8.2, 1.2 Hz); 8.50 (1H, dd, *J* = 7.8, 1.2 Hz); 8.45 (1H, d, *J* = 8.2 Hz); 8.30 (1H, d, *J* = 7.8 Hz); 7.83 (1H, t, *J* = 7.8 Hz); 7.75 (1H, td, *J* = 7.4, 1.2 Hz); 7.44–7.58 (3H, m); 7.36 (1H, d, *J* = 7.8 Hz); 7.12 (1H, d, *J* = 8.2 Hz); 7.02 (1H, td, *J* = 7.4, 1.2 Hz); FTIR (KBr) ν_max_ 3034, 1653, 1571, 1470, 1384, 1325, 1301, 1280, 1142, 993, 837, 776, 743,701 cm^-1^; EIMS *m/z* 335 (26), 334 [M]^+^ (100), 333 (57), 306 (15), 278 (10), 256 (24), 201 (22), 200 (27), 167 (12), 79 (16).

**3-[N-(Quinolin-2-ylmethylidene)amino]benzo[*de*]anthracen-7-one (2d).** The product was obtained as a light brown solid with a yield of 65%; mp 270–271 °C; ^1^H NMR (400 MHz, DMSO) δ: 8.98 (1H, s), 8.91 (1H, dd, *J* = 8.2, 1.2 Hz), 8.85 (1H, dd, *J* = 7.4, 1.2 Hz), 8.50–8.58 (3H, m), 8.32–8.28 (2H, m), 8.21 (1H, d, *J* = 8.2 Hz), 7.93 (1H, d, *J* = 8.2 Hz), 7.74-7.82 (3H, m), 7.66 (1H, td, *J* = 7.4, 1.2 Hz), 7.56 (1H, td, *J* = 7.4, 1.2 Hz), 7.45 (1H, d, *J* = 7.8 Hz); ^13^C NMR (100 MHz, DMSO) δ 188.3, 162.4, 160.2, 151.5, 150.8, 148.7, 134.5, 131.9, 131.4, 131.0, 130.3, 130.4, 129.7, 128.5, 128.2, 126.3, 126.2, 125.3, 124.4, 118.8, 115.4; FTIR (KBr) ν_max_ 3056, 1649, 1598, 1567, 1378, 1276, 1163, 1112, 824, 779, 753, 702 cm^-1^; EIMS *m/z* 246 (20), 245 (100), 217 (16), 189 (18), 109 (13), 94 (14).

**3-[N-(Thiophen-2-ylmethylidene)amino]benzo[*de*]anthracen-7-one (2e).** The product was obtained as a red-brown crystalline solid with a yield of 89%; mp 225–226 °C; ^1^H NMR (400 MHz, CDCl_3_) δ 8.80–8.84 (2H, m), 8.75 (1H, s), 8.51 (1H, dd, *J* = 7.8, 1.6 Hz), 8.45 (1H, d, *J* = 7.8 Hz), 8.32 (1H, d, *J* = 8.2 Hz), 7.81 (1H, t, *J* = 7.4 Hz), 7.74 (1H, t, *J* = 7.8), 7.58–7.64 (2H, m), 7.54 (1H, t, *J* = 7.4 Hz), 7.28 (1H, d, *J* = 7.8 Hz), 7.20 (1H, t, *J* = 4.3 Hz); ^13^C NMR (100 MHz, DMSO) δ 182.9, 150.89, 147.9, 147.8, 139.6, 137.5, 136.3, 134.4, 133.7, 132.6, 130.2, 129.9, 129.4, 128.9, 128.7, 128.5, 128.2, 127.9, 127.7, 127.4, 127.3, 127.1, 126.8, 126.1, 124.9, 124.2, 124.1, 123.1, 122.8, 114.9, 113.0; FTIR (KBr) ν_max_ 3074, 1644, 1606, 1594, 1563, 1431, 1383, 1278, 1216, 1042, 958, 837, 777, 736, 700 cm^−1^; EIMS *m/z* 340 (27), 339 [M]^+^ (100), 338 (28), 310 (12), 201 (20), 200 (21).

**3-[N-(Furan-2-ylmethylidene)amino]benzo[*de*]anthracen-7-one (2f).** The product was obtained as a dark yellow powder with a yield of 70%; mp 221–222 °C; ^1^H NMR (400 MHz, CDCl_3_) δ 8.77 (1H, dd, *J* = 8.2, 1.3 Hz), 8.73 (1H, d, *J* = 8.0 Hz), 8.64 (1H, dd, *J* = 7.3, 1.3 Hz), 8.56 (1H, d, *J* = 8.2 Hz), 8.29 (1H, dd, *J* = 7.9, 1.5 Hz), 8.02 (1H, d, *J* = 1.8 Hz), 7.88 (1H, dd, *J* = 8.2, 7.3 Hz), 7.81 (1H, ddd, *J* = 8.3, 7.1, 1.5 Hz), 7.57 (1H, ddd, *J* = 8.0, 7.1, 1.0 Hz), 7.48 (1H, d, *J* = 8.0 Hz), 7.31 (1H, dd, *J* = 3.5, 0.8 Hz), 6.75 (1H, dd, *J* = 3.5, 1.8 Hz); ^13^C NMR (100 MHz, DMSO) δ 183.2, 152.4, 150.7, 150.2, 149.6, 147.8, 136.3, 134.3, 131.8, 130.2, 128.8, 128.6, 128.1, 127.9, 127.7, 127.1, 126.8, 124.2, 124.0, 119.2, 114.7, 113.3; FTIR (KBr) ν_max_ 3041, 1646, 1622, 1567, 1477, 1383, 1011, 776, 745, 702, 591 cm^-1^; EIMS *m/z* 324 (25), 323 [M]^+^ (100), 294 (25), 200 (23), 161 (12).

General Procedure for synthesis of amines **3a**–**f**: Dissolve 0.5 mmol of the corresponding azomethine **2a**–**f** in 15–20 mL of dimethylformamide and add 0.5 mmol of NaBH_4_ in small portions over 15 min. Add 2–3 mL of methanol dropwise to the solution prepared at room temperature. Stir the reaction mixture at room temperature in an ultrasonic bath for 5–6 h, monitoring the reaction by thin layer chromatography in benzene: acetonitrile 3: 1. The product obtained is precipitated with water, filtered, dried, and recrystallized from chloroform.

**3-[N-(1*H*-Pyrrol-2-ylmethyl)amino]benzo[*de*]anthracen-7-one (3a).** The product was obtained as a brown crystalline solid with a yield of 78%; mp 224–225 °C; ^1^H NMR (400 MHz, DMSO) δ: 8.92 (1H, 1s); 8.46 (1H, dd, *J* = 7.8, 1.2 Hz); 8.31 (1H, d, *J* = 8.2 Hz); 8.17–8.23 (2H, m); 7.63-7.74 (2H, m); 7.35-7.44 (3H, m); 6.93 (2H, dd, *J* = 5.9, 2.3 Hz); 6.76 (1H, d, *J* = 8.2 Hz); 5.17 (1H, t, *J* = 5.9 Hz); 4.51 (2H, d, *J* = 4.7 Hz);^13^C NMR (100 MHz, DMSO) δ 183.0; 163.0; 147.9; 139.6; 137.6; 136.3; 133.7; 130.0; 129.7; 129.5; 128.9; 128.7; 128.5; 128.2; 127.7; 127.3; 124.8; 123.1; 122.8; 114.9; 113.1; 105.1; 46.5; FTIR (KBr) ν_max_ 3412, 3217, 3010, 2853, 1626, 1571, 1527, 1472, 1366, 1320, 1269 cm^−1^; EIMS *m/z* 323 [M-1]^+^ (22), 322 (100), 321 (95), 201 (24), 200 (23), 161 (12), 146 (12).

**3-[N-(Pyridin-4-ylmethyl)amino]benzo[*de*]anthracen-7-one (3b).** The product was obtained as a red crystalline solid with a yield of 77%; mp 240–241 °C; ^1^H-NMR (400 MHz, DMSO) δ 8.83 (1H, d, *J* = 8.2 Hz), 8.64 (1H, d, *J* = 7.4 Hz), 8.46 (2H, d, *J* = 5.1 Hz), 8.39 (1H, d, *J* = 8.2 Hz), 8.30 (1H, d, *J* = 8.2 Hz), 8.21 (1H, dd, *J* = 8.0, 1.5 Hz), 8.05 (1H, t, *J* = 6.0 Hz), 7.81 (1H, t, *J* = 8.0 Hz), 7.66 (3H, t, *J* = 7.5 Hz), 7.36–7.39 (3H, m), 6.50 (1H, d, *J* = 8.2 Hz), 4.64 (2H, d, *J* = 6.0 Hz); ^13^C NMR (100 MHz, DMSO) δ 183.2, 150.1, 148.9, 147.5, 137.5, 133.8, 130.0, 129.6, 128.8, 128.5, 128.3, 127.4, 126.3, 125.0, 124.1, 123.1, 122.9, 122.5, 122.3, 113.7, 105.2, 79.4. FTIR (KBr) ν_max_ 3388, 3076, 2929, 1636, 1566, 1534, 1462, 1317, 1267, 1128, 1011, 952, 768, 703 cm^−1^; EIMS *m/z* 335 [M-1]^+^ (28), 334 (100), 201 (24), 200 (25), 133 (26).

**3-[N-(Pyridin-2-ylmethyl)amino]benzo[*de*]anthracen-7-one (3b).** The product was obtained as a bright red powder with a yield of 70%; mp 168–170 °C; ^1^H NMR (400 MHz, DMSO) δ 8.90 (1H, d, *J* = 8.2 Hz); 8.69 (1H, dd, *J* = 7.4, 1.2 Hz); 8.45 (1H, d, *J* = 8.2 Hz); 8.24-8.37 (2H, m); 7.94 (1H, t, *J* = 5.9 Hz); 7.83 (1H, t, *J* = 7.8 Hz); 7.71 (1H, td, *J* = 7.4, 1.6 Hz); 7.42 (1H, t, *J* = 7.4 Hz); 7.19 (1H, dd, *J* = 7.8, 1.6 Hz); 7.07 (1H, td, *J* = 7.8, 1.6 Hz); 6.89 (1H, d, *J* = 7.8 Hz); 6.71 (1H, td, *J* = 7.4, 1.2 Hz); 6.60 (1H, d, *J* = 8.2 Hz); 4.57 (2H, d, *J* = 4.7 Hz); FTIR (KBr) ν_max_ 3372, 3067, 2924, 2854, 1639, 1576, 1528, 1473, 1439, 1315, 1266, 1175, 1129, 957, 763, 701, 612 cm^−1^; EIMS *m/z* 337 (27), 336 [M]^+^ (100), 258 (19), 244 (29), 217 (29), 189 (12).

**3-[N-(Quinolin-2-ylmethyl)amino]benzo[*de*]anthracen-7-one (3d).** The product was obtained as a dark red powder with a yield of 70%; mp 177–178 °C; ^1^H NMR (400 MHz, CDCl_3_) δ 8.84 (1H, dd, *J* = 7.8, 1.2 Hz), 8.50 (1H, d, *J* = 8.2 Hz), 8.45 (1H, dd, *J* = 7.8, 1.2 Hz), 8.29 (1H, d, *J* = 8.2 Hz), 8.13-8.31 (3H, m), 7.74-7.86 (3H, m), 7.54-7.66 (2H, m), 7.43 (1H, d, *J* = 8.2 Hz), 7.38 (1H, t, *J* = 7.4 Hz), 7.08 (1H, t, *J* = 3.9 Hz), 6.71 (1H, d, *J* = 8.2 Hz), 4.77 (2H, d, *J* = 4.3 Hz); FTIR (KBr) ν_max_ 3368, 3055, 1645, 1570, 1530, 1461, 1306, 1266, 1172, 761 cm^−1^; EIMS *m/z* 246 (19), 245 (100), 217 (17), 189 (18), 109 (12), 94 (10).

**3-[N-(Thiophen-2-ylmethyl)amino]benzo[*de*]anthracen-7-one (3e).** The product was obtained as a dark red powder with a yield of 72%; mp 194–195 °C; ^1^H NMR (400 MHz, DMSO) δ 8.83 (1H, dd, *J* = 8.6, 1.2 Hz), 8.68 (1H, dd, *J* = 7.4, 1.2 Hz), 8.50 (1H, d, *J* = 8.6 Hz), 8.39 (1H, d, *J* = 8.2 Hz), 8.27 (1H, dd, *J* = 7.8, 1.2 Hz), 8.06 (1H, t, *J* = 5.9 Hz), 7.83 (1H, t, *J* = 8.2 Hz), 7.73 (1H, td, *J* = 7.8, 1.2 Hz), 7.43 (1H, td, *J* = 7.8, 1.2 Hz), 7.38 (1H, dd, *J* = 5.1, 1.2 Hz), 7.17 (1H, dd, *J* = 3.5, 1.2 Hz), 6.99 (1H, t, *J* = 4.3 Hz), 6.82 (1H, d, *J* = 8.6 Hz), 4.83 (1H, d, *J* = 5.9 Hz); FTIR (KBr) ν_max_ 3442, 3098, 1633, 1569, 1527, 1474, 1312, 1262, 1172, 769, 688 cm^−1^; EIMS *m/z* 342 (10), 341 [M]^+^ (40), 244 (10), 217 (12), 97 (100).

**3-[N-(Furan-2-ylmethyl)amino]benzo[*de*]anthracen-7-one (3f).** The product was obtained as a bright red powder with a yield of 74%; mp 187-190 °C; FTIR (KBr) ν_max_ 3389, 3071, 3034, 1635, 1572, 1542, 1463, 1322, 1270, 1178, 1014, 764, 703, 612 cm^−1^; EIMS *m/z* 324 [M-1]^+^ (25), 323 (100), 322 (16), 321 (95), 294 (30), 201 (19), 200 (26), 161 (19).

### 3.2. Spectroscopic Measurements

The spectral properties of the investigated compound were measured in benzene (C_6_H_6_), chloroform (CHCl_3_), ethyl acetate (EtOAc), acetone, ethanol (EtOH), dimethyl sulfoxide (DMSO), and dimethylformamide (DMF) with concentrations of 10^−5^ M at an ambient temperature in 10 mm quartz cuvettes. All solvents were of p.a. or analytical grade. The absorption spectra were obtained using the UV-visible spectrophotometer SPECORD^®^ 80 (Analytik Jena AG, Germany). The fluorescence emission spectra were recorded on a FLSP920 (Edinburgh Instruments Ltd., Edinburgh, UK) spectrofluorometer in the visible range 450–800 nm. The quantum yields were measured relative to rhodamine 101 as standard.

### 3.3. Single Crystal X-ray Diffraction Analysis

For compounds **2a**, **2e**, and **3a**, diffraction data were collected at low temperature on an automatic four-circle diffractometer single-crystal X-ray diffractometer. The crystal structures were solved by direct methods with the ShelXT [42] structure solution program using intrinsic phasing and refined with the SHELXL refinement package [43]. All calculations were performed with the help of Olex2 software [44]. Table 5 lists the main crystal data for these compounds.

## 4. Conclusions

In the present research, synthetic method for preparing new benzanthrone 3-iminoderivatives was implemented by heating 3-aminobenzanthrone with a series of heterocyclic aldehydes. The synthesized azomethines were reduced to appropriate amines. In such a way, six new azomethines and six new amines were obtained with 65–90% yields as crystalline compounds from yellow to brown. The thermogravimetric analysis results showed that synthesized amines were thermally stable; hence, these derivatives may have various applications. The obtained compounds absorb at 420–525 nm, have relatively large Stokes shifts (up to 150 nm in ethanol), and emit at 500–660 nm. The achieved results testify that emission of the aimed dyes is sensitive to the solvent polarity showing negative fluorosolvatochromism for azomethines and positive fluorosolvatochromism for prepared amines. Fluorescence in the red region of the spectrum provides a high analytical sensitivity of the method, which further expands the possibilities of practical application of the synthesized amines.

## Figures and Tables

**Table 1:** Several geometrical parameters (Å, °) for molecules **2a**, **2e**, and **3a**.

Bond, Angle	2a	2e	3a
N18−C3	1.402(1)	1.405(2)	1.376(1)
N18−C19	1.281(2)	1.277(2)	1.458(1)
C19−C20	1.428(2)	1.439(2)	1.489(1)
C20−C21	1.383(2)	1.363(2)	1.372(2)
C21−C22	1.404(2)	1.410(2)	1.418(2)
C22−C23	1.364(2)	1.348(3)	1.362(2)
C23−S24	-	1.715(2)	-
C23−N24	1.349(2)	-	1.368(1)
S24−C20	-	1.721(1)	-
N24−C20	1.376(2)	-	1.369(2)
C3−N18−C19	119.8(1)	119.7(1)	120.44(9)
N18−C19−C20	122.2(1)	121.8(2)	111.51(9)
C19−C20−C21	128.9(1)	128.9(1)	130.6(1)
C20−C21−C22	106.9(1)	113.3(2)	107.4(1)
C21−C22−C23	107.7(2)	112.2(2)	107.3(1)
C22−C23−S24	-	112.3(1)	-
C22−C23−N24	108.8(1)	-	108.3(1)
C23−S24−C20	-	91.34(9)	-
C23−N24−C20	109.1(1)	-	109.4(1)
S24−C20−C19	-	121.6(1)	-
N24−C20−C19	123.3(1)	-	121.7(1)

**Table 2:** Absorption maxima (in nm) of obtained derivatives in various organic solvents.

Solvent	1	2a	3a	2c	3c	2e	3e	2f	3f
Benzene	465	426	478	426	487	437	479	435	475
CHCl_3_	475	430	481	442	499	441	489	443	486
EtOAc	486	422	487	422	490	433	488	432	484
Acetone	495	424	489	424	502	437	498	435	495
EtOH	519	430	517	430	515	444	520	439	514
DMF	514	428	514	429	511	443	510	442	510
DMSO	524	432	524	432	522	447	522	446	519

**Table 3:** Fluorescence maxima (in nm) of obtained derivatives in various organic solvents.

Solvent	1	2a	3a	2c	3c	2e	3e	2f	3f
Benzene	570	563	577	560	577	506	567	508	564
CHCl_3_	590	530	608	593	609	540	602	508;583	597
EtOAc	592	516;576	592	516;580	592	497	586	509;579	584
Acetone	625	530	615	526	615	518	609	526;587	607
EtOH	659	516	657	553	657	560	657	553	655
DMF	622	530	627	533	627	528	621	533	629
DMSO	634	531	640	534	637	568	634	534	632

**Table 4:** Emission quantum yields of initial compound **1** and obtained derivatives **2**–**3** in various organic solvents.

Solvent	1	2a	3a	2c	3c	2e	3e	2f	3f
Benzene	0.60	0.01	0.51	0.02	0.55	0.03	0.57	0.02	0.56
CHCl_3_	0.37	0.01	0.35	0.03	0.38	0.03	0.42	0.03	0.40
EtOAc	0.34	0.03	0.52	0.04	0.57	0.04	0.61	0.03	0.58
Acetone	0.12	0.02	0.36	0.04	0.36	0.03	0.40	0.03	0.37
EtOH	0.08	0.01	0.09	0.02	0.09	0.02	0.11	0.02	0.09
DMF	0.21	0.02	0.20	0.03	0.22	0.03	0.24	0.04	0.23
DMSO	0.26	0.02	0.24	0.03	0.25	0.03	0.27	0.03	0.25

**Table 5:** Crystal data and structure refinement parameters for compounds **2a**, **2e**, and **3a**.

Parameter	2a	2e	3a
Empirical formula	C_22_H_14_N_2_O	C_22_H_13_NOS	C_22_H_16_N_2_O·½C_6_H_6_
Formula weight	322.37	339.41	363.42
Diffractometer	Rigaku, XtaLAB Synergy, Dualflex, HyPix	Bruker-Nonius KappaCCD	Rigaku, XtaLAB Synergy, Dualflex, HyPix
Radiation	Cu-Kα (λ = 1.54184 Å)	Mo-Kα (λ = 0.71073 Å)	Cu-Kα (λ = 1.54184 Å)
Temperature (K)	130	173	150
Crystal size (mm^3^)	0.11 × 0.07 × 0.02	0.32 × 0.27 × 0.11	0.19 × 0.03 × 0.02
Crystal system	Monoclinic	Monoclinic	Monoclinic
Space group	*P*2_1_/*c*	*P*2_1_/*a*	*P*2_1_/*c*
*a* (Å)	11.4365(3)	12.9304(3)	11.8920(1)
*b* (Å)	8.1050(2)	9.2304(3)	8.19563(8)
*c* (Å)	16.5061(3)	14.4560(4)	18.9101(2)
β (°)	94.054(2)	112.567(1)	96.8959(9)
Unit cell volume (Å^3^)	1526.17(6)	1593.26(8)	1829.69(3)
Molecular multiplicity	4	4	4
Calculated density (g/cm^3^)	1.403	1.415	1.319
Absorption coefficient (mm^−^^1^)	0.691	0.212	0.636
*F*(000)	672	704	764
2θ_max_ (°)	155.0	57.0	155.0
Reflections collected	16174	4376	19931
Number of independent reflections	3200 (*R*_int_ = 0.064)	4089 (*R*_int_ = 0.024)	3879 (*R*_int_ = 0.034)
Reflections with *I* > 2σ(*I*)	2888	3157	3543
Number of refined parameters	242	238	262
Goodness of fit	1.029	1.047	1.074
*R*-factors (*R*1 for *I* > 2σ(*I*), and *wR*2 for all data)	0.0491, 0.1490	0.0497, 0.1242	0.0414, 0.1209
Δρ_max_, Δρ_min_ (e Å^−^^3^)	0.247 −0.311	0.217 −0.256	0.280 −0.182
CCDC deposition number	2062655	2062656	2062657

## Data Availability

The data presented in this study are available in this article.

## Sample Availability

Samples of the compounds are available from the authors.
